# Role of MCP-1 as an inflammatory biomarker in nephropathy

**DOI:** 10.3389/fimmu.2023.1303076

**Published:** 2024-01-04

**Authors:** Yanlong Liu, Ke Xu, Yuhua Xiang, Boyan Ma, Hailong Li, Yuan Li, Yue Shi, Shuju Li, Yan Bai

**Affiliations:** ^1^ Heilongjiang Provincial Health Commission, Harbin, China; ^2^ Heilongjiang University of Chinese Medicine, The Second Clinical Medical College, Harbin, China; ^3^ Heilongjiang Academy of Traditional Chinese Medicine, Harbin, China; ^4^ The First Affiliated Hospital of Harbin Medical University, Harbin, China

**Keywords:** MCP-1, inflammatory markers, primary nephropathy, secondary nephropathy, hereditary nephropathy

## Abstract

The Monocyte chemoattractant protein-1 (MCP-1), also referred to as chemokine ligand 2 (CCL2), belongs to the extensive chemokine family and serves as a crucial mediator of innate immunity and tissue inflammation. It has a notable impact on inflammatory conditions affecting the kidneys. Upon binding to its receptor, MCP-1 can induce lymphocytes and NK cells’ homing, migration, activation, differentiation, and development while promoting monocytes’ and macrophages’ infiltration, thereby facilitating kidney disease-related inflammation. As a biomarker for kidney disease, MCP-1 has made notable advancements in primary kidney diseases such as crescentic glomerulonephritis, chronic glomerulonephritis, primary glomerulopathy, idiopathic proteinuria glomerulopathy, acute kidney injury; secondary kidney diseases like diabetic nephropathy and lupus nephritis; hereditary kidney diseases including autosomal dominant polycystic kidney disease and sickle cell kidney disease. MCP-1 not only predicts the occurrence, progression, prognosis of the disease but is also closely associated with the severity and stage of nephropathy. When renal tissue is stimulated or experiences significant damage, the expression of MCP-1 increases, demonstrating a direct correlation with the severity of renal injury.

## 1 Introduction

The immune system plays a crucial role in the pathogenesis of kidney disease. Dysfunction of the immune system in patients with kidney disease leads to injury to renal parenchymal cells, subsequently triggering an inflammatory response. Deposition of immune complexes within the kidneys disrupts normal renal function, resulting in manifestations such as proteinuria and fibrosis (1). Chemokines are a class of small cytokines or signaling proteins secreted by cells that possess the ability to induce directional chemotaxis of nearby responsive cells. They play a crucial role in the migration of innate and adaptive immune cells and are closely associated with the initiation and maintenance of inflammatory response (2). Chemokines can be classified into four primary subfamilies, namely CXC, CC, CX3C, and XC. Monocyte chemoattractant protein-1 (MCP-1), also referred to as chemokine ligand 2 (CCL2), belongs to the CC subfamily of cytokines (3). The main producers of MCP-1 are monocytes and macrophages, although it is also expressed by various other cell types such as endothelial cells, fibroblasts, epithelial cells, smooth muscle cells, mesangial cells, astrocytes, and microglia (4, 5). Upon binding to its receptor, MCP-1 can induce homing, migration, activation, differentiation,and development of lymphocytes and NK cells; facilitate infiltration of monocytes and macrophages; promote inflammation occurrence; stimulate angiogenesis; as well as exert fibrotic effects (6). Studies have demonstrated that MCP-1 plays a significant role in fibrosis affecting multiple organs (7). When the renal tissue is stimulated, there is a notable rise in the expression level of MCP-1, which exhibits a strong positive correlation with the extent of renal injury. The primary mechanism involves MCP-1 activating monocytes through chemotaxis thereby promoting their secretion offibrogenic cytokines such as TGF-B,resulting in extracellular matrix accumulation within glomeruliand renal tubules leading to renal interstitial fibrosis,promoting glomerulosclerosis,and ultimately causing renal failure (8). Research has shown that the release of MCP-1 is controlled by both cytokines with pro-inflammatory properties and those with anti-inflammatory properties. Inflammatory cytokines, including IL-1, TNF-α, and IL-6, have the potential to augment MCP-1 secretion from renal tubular epithelial cells. On the other hand, anti-inflammatory cytokines like retinoic acid and glucocorticoids can inhibit MCP-1 secretion. Moreover, various cytokines or growth factors including IL-4, macrophage colony-stimulating factor, platelet-derived growth factor, TGF-β, lipopolysaccharide, reactive oxygen species, and immune complexes are also capable of inducing MCP-1 production and participating in the reparative processes following kidney injury (9).

Conventional clinical indicators, such as proteinuria levels, hypertension status, and decreased glomerular filtration rate (GFR), fail to effectively detect patients with substantial tubulointerstitial involvement or individuals at higher risk of accelerated disease progression (10, 11). The evaluation of interstitial fibrosis and tubular atrophy (IFTA) necessitates invasive and delayed renal biopsy, which hampers the ability to continuously monitor disease progression (12, 13). Blood urea nitrogen, GFR estimation formula, serum creatinine, and albuminuria are currently utilized to evaluate the presence and progression of diabetic nephropathy (DN). However, they lack precision and sensitivity towards minor changes in renal function (14, 15). The most dependable indicator for renal function in patients with autosomal dominant polycystic kidney disease (ADPKD) is the height corrected total kidney volume (htTKV). Nevertheless, it is relatively expensive and exhibits limited sensitivity (16, 17). Therefore, there is an urgent need to discover novel biomarkers that possess high sensitivity, specificity, and ease of operation. The role of MCP-1 as a non-invasive biomarker has garnered significant attention in research. In this context, the utilization of MCP-1 as an inflammation marker in nephropathy is summarized below.

## 2 MCP-1/CCR2 signaling axis and functional architecture

**Figure 1:**
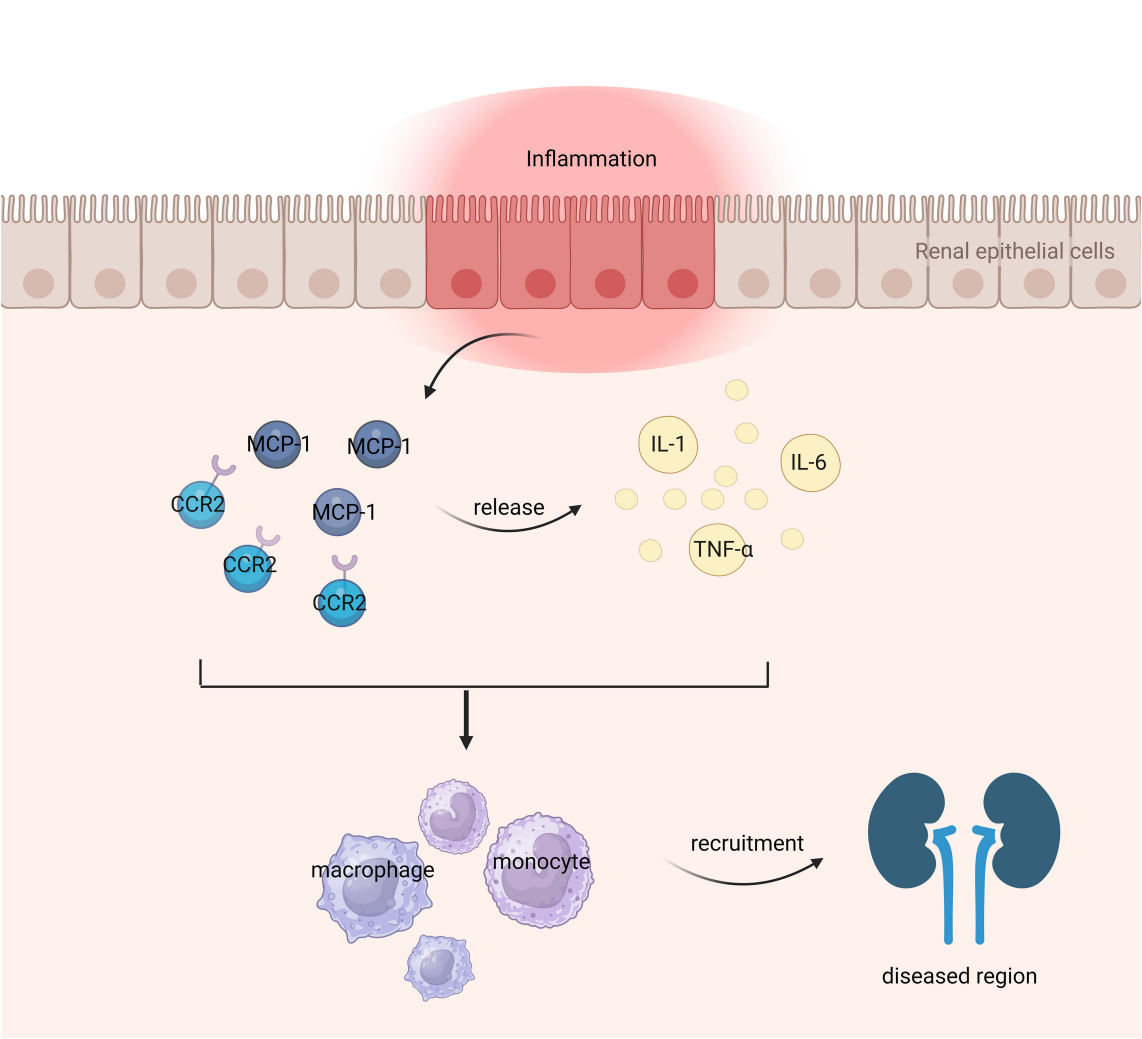
Role of MCP-1 as an inflammatory marker in nephropathy.

The MCP-1 gene is located on human chromosome 17q11.2-q21.1 (18), with a size of approximately 13 kilodaltons and consisting of 76 amino acid residues (19). Within its primary structure, there are two crucial regions for biological activity: amino acids 10 to 13 and amino acids 34 to 35 (3). Alterations in the former region result in reduced biological activity, while mutations in the latter region lead to a complete loss of MCP-1’s activity. Four conserved cysteine residues can be found at positions 11, 12, 36, and 52 within the MCP-1 protein molecule (20). These cysteine residues form disulfide bonds between them, specifically Cys11-Cys36 and Cys12-Cys52 which create a left-handed helix structure. The presence of these disulfide bonds may be essential for maintaining MCP-1’s biological function. In terms of secondary structure, MCP-1 consists of a four-stranded β-sheet along with an unstructured N-terminal loop and a C-terminal α-helix positioned above the Greek bond formed by β-folding process (21). The N-terminal segment plays a significant role in activating receptors associated with MCP-1 signaling pathway activation. Additionally, any missing residues at the N-terminal region would result in loss or reduction of its activity (21). **Figure 1** illustrates the mechanism of MCP-1 as an inflammatory marker in nephropathy.

CCR2, the major receptor of MCP-1, belongs to the G protein-coupled receptor superfamily and consists of an amino-terminal extracellular domain and seven A-helix transmembrane structures rich in hydrophobic amino acids (22). CCR2 is widely expressed in immature dendritic cells, plasmacytoid dendritic cells, basophils, helper T cells 1, helper T cells 2, natural killer cells and helper T cells 17, etc (23). CCR2, as the main receptor of MCP-1, is an important chemokine in renal fibrosis. When MCP-1 binds to CCR2, the MCP-1/CCR2 axis is activated (24). Activation of the MCP-1/CCR2 axis induces chemotaxis and activation of inflammatory cells and initiates a series of signaling cascades in renal fibrosis (25). It mediates and promotes renal fibrosis by recruiting monocytes and promotes the activation and transdifferentiation of macrophages (26). The rationale for targeting MCP-1/CCR2 to treat human related diseases is to use drugs to block MCP-1 or CCR2 and inhibit the activation and conduction of the MCP-1/CCR2 axis, thereby reducing inflammatory cells and proinflammatory cytokines. Thus, the MCP-1/CCR2 axis is also a very important player in chemokine signaling in renal fibrosis (27).

## 3 MCP-1 as a biomarker

### 3.1 Primary nephropathy

#### 3.1.1 Crescentic glomerulonephritis

In a prospective cohort study, 82 patients with pathologically confirmed crescentic glomerulonephritis (CrGN) were followed up for 5 years to investigate the role of urinary chemokines and cytokines as prognostic biomarkers in CrGN patients. This study supports the clinical significance of baseline GFR and IFTA score, while previous research has demonstrated that urinary MCP-1 (uMCP-1) can be utilized as a predictive biomarker for assessing renal prognosis in patients with CrGN (28). MCP-1 is essential for attracting macrophages, which stimulates tissue factor expression and fibrin deposition, leading to glomerular crescent formation and subsequent promotion of chronic fibrosis phase in CrGN (29). Macrophage depletion can reduce kidney injury and glomerular crescents, whereas adoptive transfer of macrophages can exacerbate crescentic glomerulonephritis in mice (30). The reduction of MCP-1 levels can result in a decrease in crescent formation, deposition of type I collagen, and renal damage in a mouse model of glomerulonephritis (31). uMCP-1 shows potential as a predictor for favorable prognosis in CrGN; however, further studies are warranted.

#### 3.1.2 Chronic glomerulonephritis

The progression of renal damage in individuals with chronic glomerulonephritis is significantly associated with the activation of the renin-angiotensin system (RAS). By evaluating the expression level of angiotensinogen (AGT) in glomerulonephritis and urinary AGT (uAGT), it becomes feasible to evaluate the extent of RAS activation within the renal region and determine the pathological state of chronic glomerulonephritis (32, 33). Hattori observed the levels of uAGT and uMCP-1 in a group of 48 children diagnosed with chronic glomerulonephritis prior to any treatment. Furthermore, they conducted an immunohistochemical analysis on AGT and CD68, while also investigating the impact of angiotensin II (Ang II) on MCP-1 expression in a separate group of 27 children who had undergone RAS blockade and received immunosuppressive agents for two years. The findings revealed a positive correlation between urinary protein level, mesangial cell proliferation score, crescent formation rate, as well as AGT and CD68 expression in renal tissue with both uAGT and uMCP-1 levels (P<0.05). In addition, the levels of uAGT and uMCP-1 showed a significant reduction after the administration of RAS blockade and immunosuppressive therapy (p<0.01). Cultured human mesangial cells (MCs) exhibited elevated MCP-1 mRNA and protein levels after Ang II treatment (p<0.01). Therefore, both uMCP-1 and uAGT can serve as valuable biomarkers for assessing the extent of glomerular injury during RAS blockade and immunosuppressive therapy among children diagnosed with chronic glomerulonephritis (34).

#### 3.1.3 Primary glomerulopathy

In a research investigation, urine specimens were obtained from individuals diagnosed with primary glomerulopathy (including IgA nephropathy, focal and segmental glomerulosclerosis, minimal change disease, membranous nephropathy) during the biopsy procedure. The value of MCP-1, EGF, and the EGF/MCP-1 ratio in predicting moderate to severe IFTA in primary glomerulonephritis was assessed using the enzyme-linked immunosorbent assay (ELISA). Univariate analysis identified connections between glomerular filtration rate, levels of EGF, the ratio of EGF to MCP-1, and IFTA. However, upon conducting multivariate analysis, it was determined that only the independent correlation between the EGF/MCP-1 ratio and IFTA remained significant. The sensitivity of the EGF/MCP-1 ratio in detecting IFTA was found to be 88%, while its specificity stood at 74% (35). Another study also came to a similar conclusion when investigating urinary biomarkers (EGF, MCP-1) and their ratio as predictors for achieving complete response in patients with biopsy-proven glomerulonephritis. The investigation also analyzed the association between these biomarkers and renal function following a period of 24 months. Complete remission (CR) was determined as the presence of urine protein levels equal to or less than 0.3 g/gCr. Out of 74 patients included in this study, 38 (51.4%) achieved CR status. The CR group exhibited significantly elevated baseline urinary levels of both EGF and the ratio of EGF/MCP-1 in comparison to the non-CR group. However, uMCP-1 did not exhibit a strong prognostic effect relative to EGF/MCP-1 (36).

#### 3.1.4 Idiopathic proteinuria glomerulopathy

A cohort of 165 individuals, including 150 females, diagnosed with idiopathic albuminuric glomerulopathy and presenting a serum concentration below 68μmol/L at the time of diagnosis, were enrolled in this study. The uMCP-1 concentrations of these patients were measured using ELISA on the day of renal biopsy, followed by subsequent follow-up. The rate of progression in end-stage renal disease (ESKD) was assessed through the application of Kaplan-Meier survival analysis. The level of uMCP-1 excretion in patients with proliferative glomerulonephritis was significantly higher compared to those with non-proliferative glomerulonephritis (p<0.001). The percentage of patients exhibiting significantly worse renal function was 0.1% in the high uMCP-1 excretion group and 9.1% in the low uMCP-1 excretion group. However, after adjusting for confounding variables such as GFR and proteinuria, there was no significant association between uMCP-1 concentration and the progression of ESKD (HR = 1.75, 95%CI = 0.64-4.75, p = 0.27). Contrary to expectations, uMCP-1 could not be considered an independent predictor for long-term outcomes in patients with idiopathic glomerulonephritis (37). Nevertheless, it should be noted that this study’s limitation lies in measuring uMCP-1 only once; thus it cannot differentiate between patients experiencing rapid remission or recurrent seizures. Further studies are warranted to repeat measurements of uMCP-1 levels throughout the course of the disease for analyzing its predictive ability regarding prognosis.

#### 3.1.5 Acute kidney injury

The baseline estimated glomerular filtration rate (eGFR) of 2,351 participants in the SPRINT study was below 60 ml/min/1.73m^2^. Exploratory factor analysis (EFA) was employed to capture distinct tubular pathophysiological processes, while a linear mixed-effect model was utilized to evaluate the association between each factor and longitudinal changes in eGFR. Cox proportional hazard regression analysis was conducted to assess the relationship between tubular factor scores and acute kidney injury (AKI). Among the 10 biomarkers examined, EFA revealed a reflection of tubular injury/fibrosis through KIM-1 and MCP-1, which were independently associated with an increased risk of AKI (HR 1.23 [1.02, 1.48]) (38). The longitudinal examination of AKI suggests that damage and inflammation may persist long after the initial insult. In a prospective cohort study involving 656 hospitalized subjects with AKI, multiple assessments of biomarkers were performed from diagnosis up to 7 months post-AKI. Cox proportional risk regression analysis was used to determine their association with a composite outcome comprising both incidence and progression of chronic kidney disease (CKD). After a follow-up period of 4.3 years, CKD and CKD progression were observed in 106 and 52 subjects respectively. Each increase in uMCP-1 levels by one standard deviation from baseline to one month was found to be significantly associated with a two- to three-fold increase in the risk of developing CKD (39). Other studies have controlled urinary concentrations for detecting biomarkers related to renal disease progression following AKI by using urinary creatinine (UCr) or urine osmolarity (UOsm) as covariates or calculating ratios such as biomarker-to-UCr ratio or biomarker-to-UOsm ratio for better prediction of outcomes. These studies have demonstrated that adjusting for UCr or UOsm strengthens the association and predictive ability for CKD when considering MCP-1 (40). The studies on MCP-1 in primary nephropathy are summarized in **Table 1**.

**Table 1:** MCP-1 as a marker of inflammation in primary nephropathy.

Research	Disease	Research Methods	Subjects	Sample Size	Source	Result
(28)	crescentic glomerulonephritis	prospective cohort study	human	36	urine and serum	urinary MCP-1, IL-10 and fractalkine are favorable prognostic markers
(34)	chronic glomerulonephritis	Clinical Research	human	48	urine	uMCP-1 and uAGT are available biomarkers
(35)	primary glomerulopathy	controlled trial	human	76	urine	EGF/MCP-1 ratio is independently associated IFTA severity
(35)	primary glomerulopathy	prospective cohort study	human	74	urine	In contrast to EGF/MCP-1, uMCP-1 did not show a strong prognostic effect
(37)	idiopathic proteinuria glomerulopathy	Long-Term Follow-Up Study	human	165	urine	Progression to ESRD in patients with idiopathic glomerulopathy was not associated with urinary MCP-1 concentration at diagnosis
(38)	acute kidney injury	Observational cohort nested in a clinical trial	human	2351	urine and serum	MCP-1 is associated with future risk for AKI
(39)	acute kidney injury	prospective cohort study	human	656	urine	Each SD increase in the change of uMCP-1 from baseline to 12 months was associated with 2- to 3-fold increased risk for CKD
(40)	acute kidney injury	prospective cohort study	human	1583	urine	The associations and predictions with CKD were significantly strengthened after indexing or adjusting for UCr or UOsm for urine kidney injury MCP-1 in patients with AKI

### 3.2 Secondary nephropathy

#### 3.2.1 Diabetic nephropathy

The presence of macroalbuminuria serves as a crucial indicator for the progression of DN (41), with most studies focusing on renal function decline or the development of ESRD in DN patients. The predictive value of uMCP-1 in determining the risk of requiring dialysis, experiencing a doubling of serum creatinine levels, or facing mortality (referred to as the primary outcome, PO) was assessed in a cohort of 56 patients with DN and type 2 diabetes mellitus (T2DM), who also had macroalbuminuria. This analysis was conducted over a follow-up period lasting approximately 30.7 ± 10 months. In Cox regression analysis, uMCP-1 levels were positively associated with the risk of PO. After adjusting for baseline albuminuria, blood pressure, and baseline creatinine, uMCP-1 (OR 11.0; 95% CI 1.6-76.4, p=0.02 for log MCP-1) remained a significant independent predictor of PO. The study concluded that uMCP-1 is independently associated with the risk of CKD progression in DN patients with macroalbuminuria. However, further investigation is needed to determine whether these biomarkers also play a role in DN patients with normoalbuminuria and microalbuminuria (42). The urinary albumin is initially filtered through the glomerulus and subsequently reabsorbed by renal tubular cells via the pathway mediated by giant protein-cubes (43). A study conducted in 2020 arrived at a similar conclusion by evaluating levels of urinary markers, such as MCP-1, in individuals with DN and analyzing their associations with eGFR and albuminuria. Urinary markers like MCP-1 exhibited significant associations with eGFR (MCP-1/Cr, p=0.023) and albuminuria (MCP-1/Cr, p<0.001). This study suggests that there is a widespread increase in urinary markers during advanced stages of DN (the extremely high risk/macroalbuminuria group), indicating their greater prominence during progressive glomerular and tubular structural abnormalities and dysfunction. Even after accounting for risk factors related to DN, the association between MCP-1/Cr and both eGFR and albuminuria remained statistically significant (44). Diabetes is recognized as the leading cause of ESKD. By examining the correlation between biomarkers indicative of tubular damage or repair, such as uMCP-1, renal function decline, and mortality rates; it was found that individuals within the highest quartile of MCP-1 had a 2.18-fold increased risk of experiencing renal function decline compared to those within the lowest quartile when considering fully adjusted models. Furthermore, for every twofold increase in baseline levels of urinary markers like MCP-1, there was an associated 10% to 40% higher risk of death (45).

The relationship between early normoalbuminuria and microalbuminuria in DN and biomarkers has been insufficiently investigated. The study included seventy-five type 2 diabetic patients with normoalbuminuria (n=25), microalbuminuria (n=25), or macroalbuminuria (n=25), as well as twenty-five healthy controls. The level of uMCP-1 was measured using ELISA. Significantly elevated levels of uMCP-1 were observed in DN patients with macroalbuminuria and microalbuminuria compared to those with normoalbuminuria and the healthy controls. Receiver operating characteristic (ROC) curve analysis was conducted to determine the optimal threshold of uMCP-1 for early diagnosis and detection of DN. The identified cut-off value was 110 pg/mg, demonstrating a sensitivity of 92% and specificity of 100%. The findings suggest that uMCP-1 holds potential as a promising novel diagnostic biomarker for the early detection of DN (46). A composite panel consisting of six biomarkers, namely osteopontin, soluble human tumor necrosis factor receptor-1, tenascin-C, vascular endothelial growth factor-a, and kidney injury molecule-1 (KIM-1), was developed using LASSO technique in a cohort of 346 normoalbuminic T2DM patients. The research findings demonstrated that a novel panel comprising six urinary biomarkers including MCP-1 effectively predicted the onset of microalbuminuria in T2DM patients with normal albuminuria (47).

The level of MCP-1 is also associated with gender, and RASS may have a significantly greater impact on interstitial lesions compared to glomerular lesions in detecting the effect of uMCP-1 on early DN (48). In this research, women exhibited a comparatively elevated initial level of uMCP-1, which displayed a more pronounced association with renal interstitial dilatation. This underscores the significance of gender as a potential risk factor for DN. Early detection of elevated uMCP-1 levels in women with T1DM may suggest a potential link between inflammatory processes and the development of renal interstitial changes during the initial stages of DN (49).

Most of the studies investigating MCP-1 and human kidney disease have primarily focused on urine, with limited research conducted on plasma or serum MCP-1 levels. uMCP-1 has been associated with unfavorable outcomes such as graft failure (50), heart disease (51), increased mortality in kidney transplant recipients (52), and impaired renal function in individuals with preserved renal function who have T2DM (53). However, there was no significant association found between serum MCP-1 concentration and decreased eGFR in T2DM patients with relatively preserved renal function (54). This study observed an elevated risk of DN progression with higher plasma MCP-1 concentrations, but only among individuals with baseline eGFR less than 45 ml/min per 1.73 m2. It is noteworthy that this study presents innovative findings regarding the association between plasma MCP-1 levels and the progression of DN in patients with moderate to severe CKD (55). Extracellular vesicles (EVs), which encompass exosomes and microvesicles, play a vital role in facilitating intercellular communication by transporting biomolecules like mRNA (56). The investigation focused on MCP-1 mRNA expression within blood EVs of patients with DN to determine its accuracy in predicting the early stages of DN. Quantitative analysis of the mRNA profile within blood EVs was conducted using qRT-PCR, while the diagnostic effectiveness of mRNA was evaluated through ROC curve analysis. A total of 196 subjects were enrolled, including 35 patients with overt DN, 53 patients with newly diagnosed DN, 62 patients with DM, and 46 healthy subjects. The predictive accuracy of MCP-1 mRNA for overt DN was determined to be at a level of 0.66 (95%CI:0.55–0.77), whereas its accuracy for predicting early-stage DN stood at a level of 0.61 (95%CI:0.51–0.71) (57).

#### 3.2.2 Lupus nephritis

A study was conducted on 20 patients with LN episodes to evaluate the association between uMCP-1 and LN severity, as well as its role as a prognostic predictor. The concentrations of uMCP-1 exhibited a significant rise in cases of acute LN (2.74 ± 0.95 ng/mg creatinine), moderate LN (1.43 ± 0.46 ng/mg creatinine), and mild LN (0.76 ± 0.57 ng/mg). There was a notable association observed between the severity of LN and the level of uMCP-1 (P<0.0358), suggesting that uMCP-1 could potentially serve as a noninvasive indicator for evaluating the grade and onset of LN. Throughout the follow-up period, 15 patients achieved either complete or partial response, resulting in a significant reduction in average uMCP-1 levels by week 8 (P<0.0001). However, for the five participants who did not respond to treatment, there was no significant alteration in average uMCP-1 levels by week 8 (P<0.4858), indicating that a lack of reduction in uMCP-1 levels at this point may indicate an unfavorable prognosis (58). The correlation coefficients and area under curve (AUC) between renal damage and uMCP-1 and tumor necrosis factor-like weak inducer of apoptosis (uTWEAK) were found to be significantly higher. The combined model of uMCP-1 and uTWEAK demonstrated an AUC of 0.887 for distinguishing active LN, with a sensitivity of 86.67% and specificity of 80.00%. For distinguishing poor prognosis LN, the AUC was 0.778, with a sensitivity of 75.00% and specificity of 81.82%. These results indicate that the combination of both biomarkers outperforms their individual use (59). Another study evaluated uMCP-1 and uTWEAK separately, revealing sensitivities for detecting active LN as follows: uTWEAK - 80.43% and 100%, while specificities were recorded at 50% and 100%, respectively; for uMCP-1 - sensitivities were observed at 82.6% and 100%, with specificities also at values of 50% and 100%, respectively (60). Furthermore, Moloi’s study concluded that during the active phase, levels of uMCP-1 are elevated but decrease after CR (61). Additionally, MCP-1 has shown potential in predicting tubulointerstitial lesions in early stages compared to traditional markers when investigating patients diagnosed with active DN through pathological biopsy analysis involving a cohort consisting of 109 LN patients along with a control group comprising 50 individuals without any kidney abnormalities. “The levels of uMCP-1 showed a marked elevation in patients diagnosed with active LN compared to those diagnosed with inactive LN (P<0.001) or individuals from the normal control group (P<<0.001) (62). Additionally, heightened levels of uMCP-l correlated positively with intensified infiltration of inflammatory cells within the interstitium, as well as increased interstitial fibrosis and tubular atrophy.

In the clinical evaluation process, quantification of proteinuria is crucial as it serves as one of the determinants for renal prognosis. The 24-hour urinary protein excretion test has traditionally been considered the “gold standard” for measuring proteinuria. However, due to its inconvenience and potential inaccuracies, many kidney disease guidelines now recommend using urinary protein/creatinine ratio (uPCR) and urinary albumin/creatinine ratio (uACR) instead (63). Relevant research has been conducted on MCP-1 as a promising candidate for biomarker identification in LN. Spearman correlation analysis was utilized to investigate the association between uMCP-1 and conventional clinical indicators. The diagnostic efficacy of uMCP-1 and uACR in assessing proteinuria levels was evaluated through ROC curve analysis. Patients with biopsy-proven LN exhibited higher levels of uMCP-1 compared to those without LN. In addition, elevated levels of both uMCP-1 and uTWEAK were observed in patients with active renal involvement (rSLEDAI ≥4). Significantly, a strong association was observed between uMCP-1 levels and the rSLEDAI score, 24-hour urinary protein excretion, and anti-double-stranded DNA antibodies in the patient cohort. Furthermore, there was a positive correlation observed between the severity of LN damage and the levels of both uMCP-1 and uTWEAK; thus suggesting that their combined use could potentially serve as a predictor for LN-associated proteinuria (64).

In relation to different pathological categories, the assessment of MCP-1 levels in various forms of LN demonstrated notably elevated urine and serum MCP-1 levels in the proliferative group compared to the non-proliferative group. The urine and serum MCP-1 levels observed in the proliferation group were 1240.65 ± 876.38 pg/ml creatinine and 354.49 ± 598.60 pg/ml creatinine, respectively. Conversely, the urine and serum MCP-1 levels noted in the non-proliferative group were 544.47 ± 430.63 pg/ml creatinine and 200.40 ± 171.83 pg/mL creatinine, correspondingly (65). The non-selective immunosuppressive drugs utilized in the clinical management of proliferative LN are associated with significant adverse effects (66). In an attempt to identify a more targeted therapeutic agent with comparable efficacy but reduced side effects, one study focused on inhibiting MCP-1 and the homeostatic chemokine stromal cell-derived factor-1 (SDF-1/CXCL12). L-enantiomeric RNA Spiegelmer^®^ chemokine antagonists, specifically MCP-1-specific mN 0X-E36 and CXCL12-specific N 0X-A12, will be administered to female MRL/lpr mice aged between 12 and 20 weeks. Research has demonstrated that simultaneous blockade of MCP-1 and CXCL12 can effectively impede the progression of proliferative LN similar to cyclophosphamide, a non-selective immunosuppressant (67).

In terms of gene prediction, a study utilizing data from gene network and GO analysis was conducted to identify candidate genes associated with LN in macrophages, revealing MCP-1’s localization in the core of the network. Further investigations have provided insights into the gene expression pattern of macrophages and revealed that macrophages derived from LN patients exhibit an upregulation of MCP-1. The Rat Genome Database (RGD) disease portal database has also indicated the association between MCP-1 and the development as well as progression of LN (68). A study conducted in Egypt aimed to investigate the potential of MCP-1 gene polymorphism as an early indicator for the development of nephropathy in patients with systemic lupus erythematosus (SLE). The findings revealed that individuals with a genotype of A/A were more prevalent among healthy controls compared to SLE patients. On the other hand, genotypes A/G (P < 0.000) and G/G (P < 0.000) were found to be more common among SLE patients than in the control group. It was observed that carriers of the G allele at MCP-1-2518 polymorphism had a significantly higher risk, over seven times, for developing nephropathy within the SLE patient population. Furthermore, patients with A/G and G/G genotypes exhibited notably elevated levels of MCP-1 when compared to those with an A/A genotype. Both MCP-1A (–2518) G gene polymorphism and increased levels of MCP-1 are believed to play crucial roles in the occurrence and progression specifically of SLE-associated nephropathy within Egypt (69). In a meta-analysis conducted in 2017 assessing the association between MCP-1 -2518A/G polymorphism and LN risk, a total sample size consisting of 1867 LN cases from 961 published case-control studies along with 10 control groups were included. The findings indicated a higher susceptibility to LN in individuals with the MCP-1 -2518A/G polymorphism. However, when stratified by ethnicity, no significant association was observed within European or Asian populations but rather predominantly found within US population due to potential genetic background variations as well as environmental exposures (70).

For comparison between urine and blood, Gupta measured serum MCP-1 and uMCP-1 in patients with ELISA. Urinary creatinine excretion values were standardized. Baseline uMCP-1 was significantly higher in active nephritis (AN) compared with active disease without nephritis (ANR), inactive disease (ID), healthy subjects (HC), and rheumatoid arthritis (RA) (p<0.001), but did not differ from DN and showed a good correlation with rSLEDAI and SLEDAI (r = 0.52 and 0.47, p<0.001), but not correlated with serum MCP-1 levels. uMCP-1 performed better than serum MCP-1, anti-dsdna antibody, C3, and C4 in ROC analysis to distinguish active nephritis from active disease without nephritis. uMCP-1 but not serum MCP-1 decreased significantly (p<0.001) (71). The 2022 study marks the initial exploration into the durability of emerging urinary biomarkers for LN, with a specific focus on assessing the renal activity index for lupus (RAIL). This comprehensive index incorporates MCP-1, KIM-1, ceruloplasmin, adiponectin, neutrophil gelatinase-associated lipocalin (NGAL), and blood phosphate. The findings demonstrate that urine biomarkers stored at -80°C for a duration of 3 months or at either 4 or 25°C for a period of 48 hours followed by storage at -80°C exhibit comparable results to freshly collected urine samples. Regardless of the conditions examined, there was no degradation in signal quality observed when exposed to dry or wet ice, or when subjected to two freeze-thaw cycles. The Spearman correlation coefficients indicated a high level of concordance. These findings suggest that RAIL biomarkers exhibit consistent stability even following brief storage under conditions relevant to clinical settings, and are capable of enduring transportation, extended storage periods, as well as multiple freeze-thaw cycles prior to bulk measurements. **Table 2** summarizes the studies of MCP-1 in secondary nephropathy.

**Table 2:** MCP-1 as a marker of inflammation in secondary nephropathy.

Research	Disease	Research Methods	Subjects	Sample Size	Source	Result
(42)	Diabetic nephropathy	double-blind placebo-controlled randomized clinical trial	human	56	urine	Urinary MCP-1 and RBP are independently related to the risk of CKD progression in patients with macroalbuminuric DN
(44)	Diabetic nephropathy	cohort study	human	185	urine	The level of MCP-1 increased with the severity of diabetic nephropathy
(45)	Diabetic nephropathy	double-blinded, randomized, controlled trial	human	712	urine	Higher baseline levels of MCP-1 (per 2-fold higher) were independently associated with 10–40% higher risk of mortality
(46)	Diabetic nephropathy	rosssectional study	human	100	urine	uMCP-1was 110 pg/mg with 92% sensitivity and 100% specificity
(47)	Diabetic nephropathy	cohort study	human	346	urine	The panel consisting of six novel urinary biomarkers effectively predicted incident microalbuminuria in people with type 2 diabetes
(49)	Diabetic nephropathy	multicenter clinical trial	human	224	urine	Elevated uMCP-1 concentration measured before clinical findings of DN in women with T1D was associated with changes in kidney interstitial volume
(53)	Diabetic nephropathy	nested case-control design	human	190	urine	Urinary monocyte chemotactic protein-1-to-creatinine ratio concentrations were strongly associated with sustained renal decline in patients with T2DM with preserved renal function
(55)	Diabetic nephropathy	cohort study	human	894	plasma	Higher plasma levels of MCP-1 was associated with increased risk of progression of DN
(57)	Diabetic nephropathy	controlled trial	human	196	blood EVs	MCP-1 mRNAs expression in blood EVs could serve as diagnostic biomarkers for early-stage DN
(58)	Lupus nephritis	controlled trial	human	40	urine	uMCP-1 could be used as a non-invasive marker for the judgement of lupus flare and lupus nephritis class
(59)	Lupus nephritis	controlled trial	human	90	urine	The combined model of uMCP-1 and uTWEAK was superior to any marker used alone
(60)	Lupus nephritis	controlled trial	human	114	urine	uMCP-1 and uTWEAK have high sensitivity and specificity in detecting active LN
(61)	Lupus nephritis	prospective observational study	human	20	urine	uMCP-1 and uTWEAK are biomarkers for active LN
(62)	Lupus nephritis	controlled trial	human	159	urine	uMCP-1 level in patients with LN is correlated with renal injury indicators
(64)	Lupus nephritis	controlled trial	human	69	urine	uMCP-1 can be used as a potential predictor of proteinuria in LN
(65)	Lupus nephritis	controlled trial	human	178	urine and serum	MCP-1 may be associated with different pathological types of LN
(67)	Lupus nephritis	controlled trial	human	120	serum	Dual blockade of CCL 2 and CXCL12 can inhibit the progression of proliferative LN
(69)	Lupus nephritis	controlled trial	human	220	urine and serum	carriers of G allele of the MCP-1 2518 polymorphism had more than 7 fold increased risk to develop glomerulo-nephropathy in patients with SLE
(71)	Lupus nephritis	controlled trial	human	121	urine and serum	uMCP-1 correlates well with LN disease activity and helps differentiate between AN and ANR patients. Its levels fall with treatment and may have a potential to predict poor response and relapse of LN

### 3.3 Hereditary nephropathy

#### 3.3.1 Autosomal dominant polycystic kidney disease

The urinary biomarkers uMCP-1, KIM-1, immunoglobulin G, 24-hour urinary albumin, β2-microglobulin (β2MG), heart-type fatty acid binding protein, and NGAL were evaluated at the beginning of the study. The changes in eGFR for each participant over time were calculated using mixed-model analysis. After accounting for age, gender, and initial htTKV levels, all indicators of urinary impairment and inflammation demonstrated correlations with baseline eGFR. Subsequent backward analysis identified uMCP-1 and β2MG as the most strongly associated factors with accelerated disease progression. When the participants were divided into three groups based on levels of uMCP-1 and β2MG, the urine biomarker score showed a stronger predictive value compared to Mayo htTKV classification (area under the curve [AUC] 0.73 [0.64-0.82] vs. 0.61 [0.51-0.71], p = 0.04). Similar to the PROPKD score (AUC 0.73 [0.64-0.82] vs.65 [.55-.75], p =0.18), these findings suggest that proximal tubules and inflammation contribute to the pathophysiology of ADPKD. Furthermore, this urine marker is more user-friendly than traditional markers (72). Another study enrolled 130 patients with ADPKD, 55 patients with renal vascular sclerosis, and 40 patients with non-ischemic CKD. The study revealed a significant upregulation of uMCP-1 under ischemic conditions. In univariate analysis, htTKV emerged as the most reliable predictor of eGFR slope variability. However, a multivariate model incorporating uMCP-1, VEGF, and β2MG levels demonstrated an enhanced ability to predict decreased eGFR in ADPKD patients compared to htTKV alone. Urinary levels of molecules associated with renal ischemia (VEGF and MCP-1) or tubular injury (β2MG) were correlated with deteriorating renal function in ADPKD patients, thus suggesting their potential as biomarkers for monitoring disease progression (73). Additionally, another study identified associations between uMCP-1 and β2MG levels and annual changes in eGFR even after adjusting for traditional risk markers (standardized β = -0.35, P = 0.001; standardized β = -0.29,P = 0.009). Incorporating uMCP-1 and β2MG into the model containing traditional risk markers significantly improved its performance (final R2 = 0.152 vs. 0.292, P = 0.001). Therefore, uMCP-1 and β2MG levels are independently linked to decreased GFR in ADPKD patients and offer greater predictive value than conventional risk indicators (74). Furthermore, several studies have assessed the urinary concentrations of 28 biomarkers in ADPKD patients while gene expression analysis has been conducted on kidneys from DBA/2FG-pcy mice alongside urine samples from these mice to evaluate the efficacy of biomarkers.The findings indicated that out of the prospective urinary biomarkers examined, twelve exhibited statistical significance with high specificity observed for uMCP-1.Moreover,the content of uMCP-1 was significantly elevated in urine samples from DBA/2FG-pcy mice compared to wild-type mice,suggesting its potential utility as a urinary biomarker for ADPKD (75).

MCP-1 may facilitate the expansion of renal cysts in ADPKD patients with PKD1 or PKD2 mutations by promoting macrophage-mediated processes. The abnormal accumulation of macrophages around the cysts promotes their growth (76). In order to explore the potential contribution of MCP-1 and macrophages in facilitating cyst expansion, a study was conducted where Pkd1 knockout alone (single knockout) or both Pkd1 and MCP-1 were knocked out in mouse renal tubules. Upregulation of MCP-1 preceded the infiltration of macrophages in single-gene knockout mice. Initially, macrophages induce a proinflammatory response and cause damage to renal tubular cells, leading to oxidative DNA damage, morphological flattening, and proliferation-dependent cystic expansion within 0-2 weeks after induction. At 2-6 weeks after induction, macrophages switch to an alternative activation phenotype that further promotes cyst growth by increasing the rate of renal tubular cell proliferation threefold more than before. In double knockout mice, reduced expression of MCP-1 and fewer numbers of macrophages resulted in less initial tubular cell damage, slower cyst growth, and improved renal function. The upregulation of MCP-1 following Pkd1 knockdown promotes the accumulation of macrophages and subsequent cyst growth through a mechanism dependent on cellular proliferation (77). The objective of this research was to examine how the absence of MCP-1 affects the concentration of macrophages in the kidneys and the progression of disease in a mouse model with congenital polycystic kidney (Cpk). To achieve this, a genetic knockout of MCP-1 was generated. The results revealed that Cpk mice exhibited rapid enlargement of renal cysts, leading to decreased renal function and mortality by postnatal day 21. However, the genetic knockdown of MCP-1 extended the survival rate, with some mice living for more than 3 months. Notably, the MCP-1 genetic knockout effectively prevented the development of pulmonary edema observed in Cpk mice and also facilitated a decrease in resting heart rate along with an increase in heart rate variability in both Cpk and non-cystic mice. These findings suggest that besides its role as a macrophage chemoattractant (78), MCP-1 plays a significant role in altering cardiac and lung function while promoting mortality in this mouse model of ADPKD.

#### 3.3.2 Sickle cell kidney disease

In a cross-sectional study of 213 children with sickle cell disease (SCD), the researchers discovered that the presence of glomerular damage was associated with elevated levels of inflammatory biomarkers, such as uMCP-1. Additionally, a short-term prospective observational cohort study involving 89 children was conducted to assess the predictive value of changes in urinary inflammatory biomarkers like MCP-1 over time for the development of glomerular proteinuria. The findings suggest that inflammatory molecules may play a crucial role in both the progression and onset of kidney disease in pediatric patients with SCD. Patients exhibiting albuminuria had significantly higher levels of inflammatory biomarkers, including MCP-1, compared to those without albuminuria. Correlation analysis revealed a significant positive association between the albumin/creatinine ratio and inflammatory biomarkers like MCP-1. These findings have important implications for understanding inflammation’s involvement in kidney disease among children with SCD and identifying potential therapeutic targets (79). Furthermore, MCP-1, which is potentially indicative of kidney damage in sickle cell patients, has also been linked to oxidative stress status. This study assessed blood and urine samples to evaluate MCP-1 levels and their correlation with malondialdehyde, a product resulting from lipid peroxidation. The results demonstrated significantly elevated levels of MCP-1 in SCD patients along with a positive correlation between MCP-1 and malondialdehyde levels, suggesting its potential as a biomarker for kidney damage associated with SCD as well as reflecting oxidative stress-induced harm present within this condition. Consequently, this research provides valuable insights into diagnosing and treating kidney damage related to SCD (80, 81). **Table 3** summarizes the studies of MCP-1 in hereditary nephropathy.

**Table 3:** MCP-1 as a marker of inflammation in hereditary nephropathy.

Research	Disease	Research Methods	Subjects	Sample Size	Source	Result
(72)	Autosomal dominant polycystic kidney disease	prospective randomized controlled trial	human	302	urine	uMCP-1 can screen ADPKD patients with rapid disease progression
(73)	Autosomal dominant polycystic kidney disease	controlled trial	human	225	urine	uMCP-1 is associated with deterioration of renal function in patients with ADPKD
(74)	Autosomal dominant polycystic kidney disease	controlled trial	human	104	urine	uMCP-1 excretion were both associated with GFR decline in ADPKD
(75)	Autosomal dominant polycystic kidney disease	gene expression analysis	human and mice	huaman(29),mice(2)	urine	uMCP-1 can be a potential common biomarker for human and murine ADPKD
(77)	Autosomal dominant polycystic kidney disease	Animal experiments	mice	Not explicitly mentioned	gene	MCP-1 was upregulated after Pkd1 knockdown
(78)	Autosomal dominant polycystic kidney disease	Animal experiments	mice	Not explicitly mentioned	gene	MCP-1 altered cardiac/pulmonary function
(79)	Sickle cell kidney disease	prospective cohort study	human	213	urine	Albumin/creatinine ratio was significantly positively correlated with MCP-1
(80)	Sickle cell kidney disease	prospective cohort study	human	50	urine	MCP-1 can also reflect damage caused by oxidative stress present in SCD.

## 4 Conclusion

The investigation of MCP-1 as a biomarker in primary nephropathy is limited, primarily encompassing certain glomerular diseases; however, it has demonstrated significant value in crescentic glomerulonephritis,chronic glomerulonephritis and AKI. Based on current research findings, MCP-1 exhibits potential utility in primary nephropathy; nevertheless, its specificity and sensitivity are relatively low, rendering it unsuitable for independent prediction.

In terms of secondary nephropathy, the research on MCP-1 primarily focuses on DN and LN. MCP-1 has been extensively investigated in various stages of DN, including early stage, progression, and ESKD. Nevertheless, early prediction remains understudied and the majority of research has centered on uMCP-1 as opposed to blood MCP-1. Additionally, gender correlation has been observed in DN. Regarding LN, MCP-1 has been well-studied and shows significant correlation with disease severity and prognosis. It exhibits increased levels during active stages and decreased levels during remission stages. Moreover, increased MCP-1 levels are linked to the infiltration of inflammatory cells in the interstitial space, fibrosis in the interstitium, and atrophy of tubules. Notably, compared to traditional markers for prediction purposes in this context. uMCP-1 can serve as a promising predictor of proteinuria in LN, with higher levels observed in proliferative LN. In terms of the genetic aspect, MCP-1-2518A/G is closely associated with the pathogenesis of LN. Additionally, uMCP-1 exhibits excellent storage stability and holds great potential as a biomarker.

In the context of hereditary kidney diseases, the level of uMCP-1 is associated with a decline in GFR among patients with ADPKD, and it exhibits superior predictive value compared to traditional risk indicators while also being more operationally convenient. Within the realm of ADPKD, MCP-1 facilitates macrophage accumulation and cyst growth through a proliferation-dependent mechanism, thereby contributing to cardiac lesions, pulmonary edema, and mortality. Patients with SCD often have glomerular and tubular dysfunction, and the emergence of some new non-invasive urine biomarkers, such as MCP-1, provides hope for early diagnosis and treatment strategies. Biomarkers expand the methods and indicators for the assessment of renal function in SCD, and provide a new way for early diagnosis and treatment. The practical significance is that it provides a non-invasive, simple, and reproducible method for renal function assessment, which helps to improve the clinical management and treatment outcomes of patients with SCD.

## Author contributions

YaL: Writing – original draft. KX: Writing – original draft. YX: Investigation, Writing – review & editing. BM: Investigation, Writing – review & editing. HL: Supervision, Writing – review & editing. YuL: Data curation, Writing – review & editing. YS: Data curation, Writing – review & editing. SL: Writing – review & editing, Writing – original draft. YB: Writing – review & editing, Writing – original draft.
